# Human histone deacetylase 6 shows strong preference for tubulin dimers over assembled microtubules

**DOI:** 10.1038/s41598-017-11739-3

**Published:** 2017-09-14

**Authors:** Lubica Skultetyova, Kseniya Ustinova, Zsofia Kutil, Zora Novakova, Jiri Pavlicek, Jana Mikesova, Dalibor Trapl, Petra Baranova, Barbora Havlinova, Martin Hubalek, Zdenek Lansky, Cyril Barinka

**Affiliations:** 1 Institute of Biotechnology CAS, BIOCEV, Prumyslova 595, 252 50 Vestec, Czech Republic; 2 Department of Biochemistry, Faculty of Natural Science, Charles University, Albertov 6, Prague 2, Czech Republic; 3 Institute of Organic Chemistry and Biochemistry of the Academy of Sciences of the Czech Republic, Flemingovo n. 2, 166 10 Prague 6, Czech Republic

## Abstract

Human histone deacetylase 6 (HDAC6) is the major deacetylase responsible for removing the acetyl group from Lys40 of α-tubulin (αK40), which is located lumenally in polymerized microtubules. Here, we provide a detailed kinetic analysis of tubulin deacetylation and HDAC6/microtubule interactions using individual purified components. Our data unequivocally show that free tubulin dimers represent the preferred HDAC6 substrate, with a *K*_*M*_ value of 0.23 µM and a deacetylation rate over 1,500-fold higher than that of assembled microtubules. We attribute the lower deacetylation rate of microtubules to both longitudinal and lateral lattice interactions within tubulin polymers. Using TIRF microscopy, we directly visualized stochastic binding of HDAC6 to assembled microtubules without any detectable preferential binding to microtubule tips. Likewise, indirect immunofluorescence microscopy revealed that microtubule deacetylation by HDAC6 is carried out stochastically along the whole microtubule length, rather than from the open extremities. Our data thus complement prior studies on tubulin acetylation and further strengthen the rationale for the correlation between tubulin acetylation and microtubule age.

## Introduction

Tubulin post-translational modifications (PTMs) provide mechanisms enabling highly conserved α/β-tubulin dimers to form microtubules (MTs) endowed with different functional properties. The best studied PTMs, collectively called the “tubulin code”, include detyrosination, glutamylation, glycylation, polyamination, phosphorylation, and acetylation^1–6^. Whereas the majority of PTMs are found within the unstructured C-terminal tubulin tails decorating the outer surface of microtubules, acetylation at the Lys40 side chain of the α-tubulin subunit (αK40) stands out due to its presumed localization within the microtubule lumen^7^. This αK40 acetylation is a hallmark of long-lived stable microtubules and can affect sperm motility, fertility, cell signaling, and cell cycle progression^8–10^. Additionally, microtubule acetylation may be a regulatory step for intracellular kinesin/dynein-mediated transport^11–13^ and, consequently, has been implicated in the pathologies of a variety of human neurodegenerative diseases. At the same time, intraluminal positioning of the flexible loop harboring αK40 understandably brings about questions concerning the accessibility of this site for relevant modifying enzymes.

The acetylation status of αK40 is defined by the opposing activities of tubulin acetyl transferases, most notably the mammalian α-tubulin N-acetyltransferase 1 (αTAT1) and its ortholog MEC-17 from *C. elegans* ^9, 14^, and lysine deacetylases, namely histone deacetylase 6 (HDAC6) and SIRT2^15–17^. Although HDAC6 and SIRT2 may interact and function together, several studies indicate that HDAC6, not SIRT2, is the major tubulin deacetylase^18, 19^. A recent report^20^ suggests that the two deacetylases might have both overlapping and distinct tubulin deacetylase activities depending on the specific structural contexts of αK40, as well as the physiological state of the cell. Of note, several additional acetylation sites in tubulin have been identified to date^21–23^, but their functional role and the characterization of the enzymes responsible require further research in this direction.

HDAC6, a member of the class IIb zinc-dependent histone deacetylases, stands out as a structurally and functionally unique member of the HDAC family due to its complex domain organization and atypical predominantly cytosolic localization. HDAC6 harbors the tandem catalytic domains DD1 and DD2, a cytoplasm-anchoring serine/glutamate-rich repeat motifs, and the C-terminal ubiquitin binding domain, which is implicated in sequestering misfolded polyubiquitinated protein aggregates and transporting them to the aggresome^24^. Several post-translational modifications, including phosphorylation, acetylation, and S-nitrosylation, have been shown to regulate HDAC6 deacetylase activity, nucleus/cytoplasm shuttling, and interactions with physiological partners^25–28^. Given the broad repertoire of HDAC6 substrates and interaction partners (e.g., heat-shock protein 90, cortactin, peroxiredoxin, β-catenin, dynein)^29–33^, it is not surprising that the enzyme is involved in many (patho)physiological processes, including cell motility and metastasis, cell signaling, protein folding and degradation, and inflammation. Consequently, targeting HDAC6 is a viable strategy for the treatment of various disorders, such as neurodegenerative diseases, multiple myelomas, and solid malignancies^34–37^.

Despite the fact that HDAC6 was identified as the major tubulin deacetylase more than 10 years ago, there is a surprising nearly total absence of qualitative/quantitative data concerning HDAC6 preferences for different tubulin forms. Moreover, when available, these findings are relatively contentious and somewhat difficult to reconcile. Finally, the majority of studies have used either partially purified HDAC6 and/or tubulin preparations, or orthologs and truncated variants of human HDAC6, as these are more readily available in the amounts required for biochemical/structural studies. In their seminal paper, Hubbert *et al*., described for the first time the way in which HDAC6 functions as a tubulin deacetylase. *In vitro* experiments using mouse HDAC6 revealed that the enzyme deacetylates assembled microtubules, but not free tubulin^15^. This notion was subsequently challenged by several reports showing that both free heterodimers and assembled MTs can serve as HDAC6 substrates, but no quantitative data to evaluate substrate preferences were presented^16, 17, 38^. In this report, we exploited a bottom-up biochemical approach using purified full-length human HDAC6 and tubulin to assess HDAC6 substrate preferences and shed light on the structural features that govern HDAC6/tubulin interactions. We also directly visualized HDAC6/tubulin interactions, suggesting that the enzyme binds preferentially to the external face of assembled microtubules.

## Results

### Expression and characterization of full-length human HDAC6

**Figure 1:**
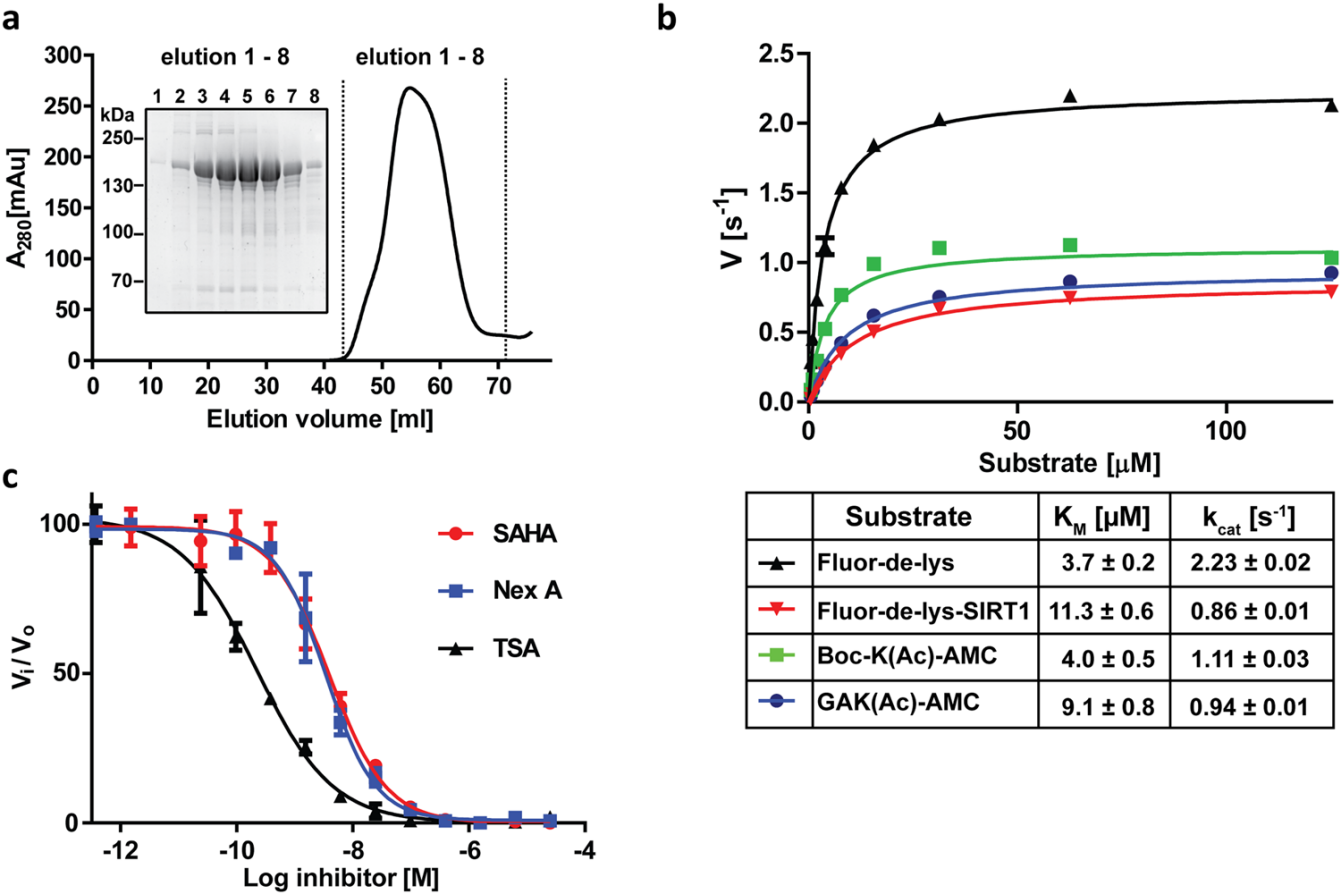
Purification and characterization of full length HDAC6. (**a**) Elution profile of human HDAC6 from a Superdex 16/600 HR200 size-exclusion column (SEC) and reducing SDS-PAGE analysis of HDAC6 fractions from the SEC, documenting monodispersity and >95% purity of the final enzyme preparation, respectively. (**b**) Steady-state kinetics of HDAC6 on commercial fluorogenic peptide substrates GAK(Ac)-AMC, Boc-K(Ac)-AMC, FLUOR-DE-LYS, and FLUOR-DE-LYS-SIRT1. Michaelis-Menten constants (K_M_ and *k*_*cat*_) for individual peptides, calculated from non-linear regression fit using the GraphPad program, are shown in the embedded table. Data represent mean values ± s.d. (n = 3). **(c)** IC5﻿0 values for SAHA (3.8 nM), Nexturastat A (2.9 nM), and Trichostatin A (0.16 nM) were determined using a fluorometric assay with 10 µM (Ac)GAK(Ac)-AMC as a substrate. Data are plotted as mean values ± s.d. from three independent experiments (n = 3).﻿

The HEK293T-based mammalian system was selected for heterologous HDAC6 expression to secure the closest semblance to the native wild-type HDAC6 protein existing in human cells/tissues. Using a combination of affinity and size-exclusion chromatography, we were able to obtain homogenous preparation of untagged human HDAC6 (Fig. 1a) with yields of approximately 2 mgs per liter of original cell culture.

Previous reports have pointed out the influence of PTMs (namely acetylation and phosphorylation) on HDAC6 deacetylase activity^25, 26, 39–41^. To determine whether any such “activity-altering” PTMs existed in our HDAC6 preparation, the protein was analyzed using liquid chromatography-tandem mass spectrometry (LC-MS/MS). The deconvolution of the MS/MS spectra revealed the absence of any known (or unreported) PTM, thus excluding the possibility of our *in vitro* experiments being impacted by HDAC6 PTMs (data not shown).

To assess the deacetylase activity of untagged HDAC6, we determined its kinetic parameters in comparison to several commercially available substrates (Fig. 1b). K_M_ values were in the low micromolar range (3.7–11.3 µM) and turnover numbers (*k*_*cat*_) ranged from 0.9–2.2 s^−1^, in good agreement with data reported in the literature^42, 43^. Furthermore, using the same substrate set, we also verified that the attachment of N-terminal tags (either HALO- or GFP-tags) does not influence HDAC6 deacetylation activity (Supplementary Fig. S1). Finally, HDAC6 was profiled against suberoylanilide hydroxamic acid (SAHA), trichostatin (TSA), and nexturastat A (Nex A), with IC_50_ values of 3.8, 0.16, and 2.9 nM, respectively, also in the range reported in scientific literature^44–47^ (Fig. 1c).

Overall, the above-mentioned characteristics confirm that our HDAC6 is a homogenous, properly folded, highly-active full-length enzyme well suited for the presented biochemical experiments.

### HDAC6 prefers tubulin dimers to microtubules

Several earlier studies assessed the deacetylation activity of HDAC6 on tubulin and polymerized microtubules^15–17, 38, 48^; however, these studies predominantly used orthologs of human HDAC6 (mouse, zebrafish), their truncated (or tagged and modified) variants, and/or unpurified (or partly purified) assay components. To the best of our knowledge, none of the prior studies attempted to quantify deacetylase preferences of the full-length human HDAC6 for free tubulin versus assembled MTs. For our biochemical experiments, we took advantage of the fact that tubulin isolated from porcine brain tissue is highly acetylated, with the αK40 acetylation levels comprising 36% of the total tubulin (Supplementary Fig. S2).

**Figure 2:**
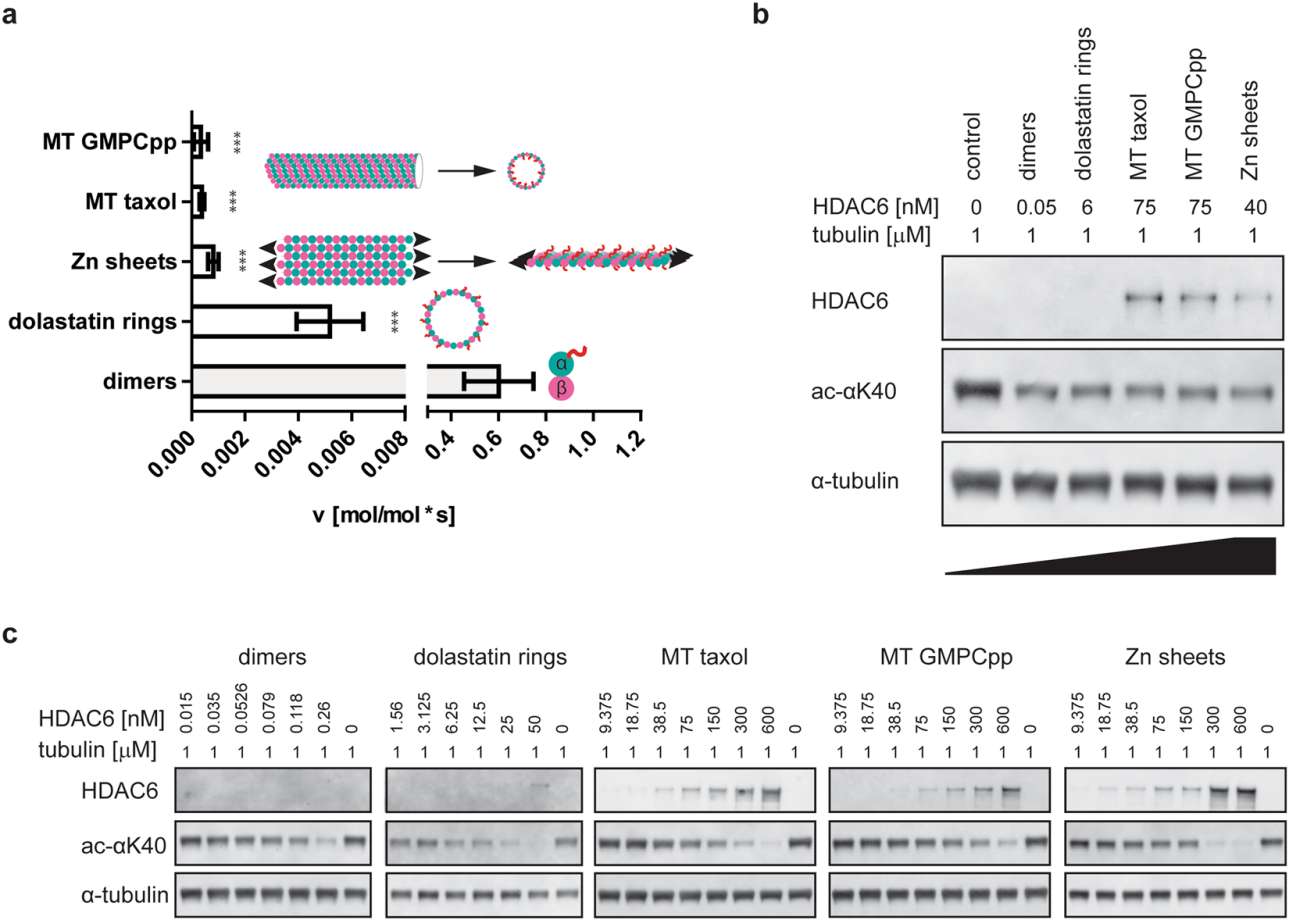
HDAC6 shows strong preference for tubulin dimers over polymeric forms. (**a**) Deacetylation activity of HDAC6 on tubulin polymers (zinc sheets, Dolastatin-10 rings, and taxol/GMPCPP-stabilized microtubules [MT]) and tubulin dimers was determined using Western blot quantification. Results clearly show that free dimers are deacetylated 100- to 1,500-fold more efficiently than are tubulin polymeric forms. Data represent mean values ± s.d. (n ≥ 3; ***p < 0.001). Organization of tubulin in polymers is illustrated in schemes embedded in the graph, with α-tubulin and β-tubulin shown as blue and pink spheres, respectively, and the acetyl group as a red ribbon. (**b**,**c**) Representative Western blots illustrating distinct HDAC6 deacetylase activity on tubulin dimers and polymeric tubulin forms. Individual substrates (1 µM) were incubated with indicated concentrations of HDAC6 for 1 h at 37 °C. Protein samples were separated by SDS-PAGE, electrotransferred to a polyvinylidene difluoride (PVDF) membrane and acetylation levels quantified using an αK(Ac)40-specific fluorescence signal normalized to the amount of total tubulin detected by rabbit polyclonal anti-α tubulin antibody.

We directly compared the catalytic activity of human HDAC6 on free tubulin dimers against its activity on paclitaxel- and GMPCPP-stabilized MTs (Fig. 2). Using highly-purified components, we showed that free tubulin dimers are the preferred HDAC6 substrate. At a 1 µM substrate concentration, the deacetylation rate was 0.6 mol/mol*s and 0.0004 mol/mol*s for tubulin dimers and stabilized MTs, respectively; in other words, the deacetylation rate was approximately 1,500-fold higher for αβ-tubulin dimers. Importantly, deacetylation rates on both paclitaxel- and GMPCPP-stabilized MTs were virtually identical, suggesting that substantially slower deacetylation of assembled MTs does not result from the compound interference, but is rather linked to either tubulin lattice packing or the limited accessibility of the luminal αK40 loop.

### Contacts within polymer lattices inhibit tubulin deacetylation by HDAC6

Unlike other known microtubule PTMs, which are positioned at the external surface of MTs, αK40 is confined to the MT lumen. Consequently, the substantially lower MT deacetylation rates (as compared to tubulin dimers) could simply result from hindered accessibility of the αK40 loop by the enzyme. Additional factors, such as the transition from curved (free tubulin) to straight (microtubules) tubulin dimer conformations or the blockage of HDAC6/tubulin interaction interfaces by longitudinal/lateral contacts within the polymer lattice, may also play a role. To distinguish between the above-mentioned scenarios, we assessed HDAC6 deacetylation activity using microtubules, Zn-sheets, and Dolastatin-10 rings, three tubulin polymeric forms with distinctly different geometries (Fig. 2).

Dolastatin-10 induces single-walled tubulin rings by curving individual protofilaments such that their luminal surface becomes accessible on the exterior of the ring^49^. In Zn-sheets, protofilaments of alternating polarity are arrayed in parallel and the “luminal” surface, as would be defined in microtubules, is exposed to the solution^50^. All tubulin polymers were deacetylated substantially (100- to 1,500-fold) slower than were free dimers (Fig. 2). As the “luminal” surface harboring the αK40 loop is exposed in both Zn-sheets and Dolastatin-10 rings, lower deacetylation rates cannot be simply attributed to the inaccessibility of the αK40 within the lumen of assembled MTs. Instead, we argue that it is the presence of both lateral and longitudinal contacts within the tubulin polymer lattices that negatively influences deacetylation by HDAC6, as documented by 100- and 750-fold slower deacetylation rates observed for Dolastatin-10 rings and Zn-sheets, respectively.

### Structural features outside the αK40 loop of αβ-tubulin are important for HDAC6 interactions

We first turned our attention to HDAC6 recognition of the isolated αK40 loop to answer the following questions: (i) is the “isolated” sequence of the αK40 loop recognized by HDAC6; (ii) what is the minimum sequence length to be processed/recognized; and (iii) is there a difference in deacetylation rates between the αK40 loop in the form of a free peptide and within the context of an αβ-tubulin dimer?

**Figure 3:**
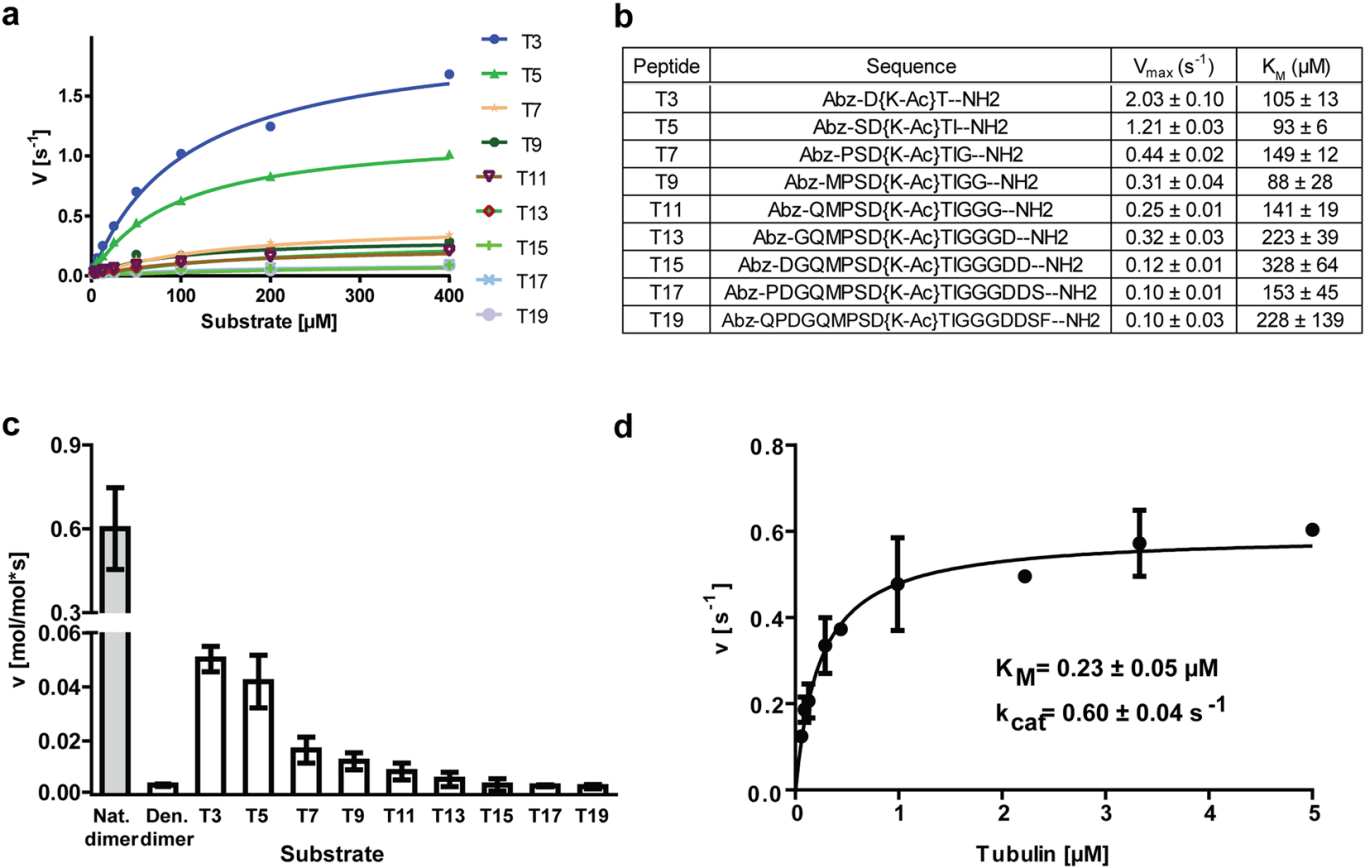
Structural features outside the αK40 loop are required for efficient substrate deacetylation by HDAC6. (**a**,**b**) Michaelis-Menten kinetics for peptides derived from the Lys40 of α-tubulin (αK40) loop (3-mer to 19-mer; T3 through T19) were determined *in vitro* using an HPLC-based assay (**a**). Corresponding kinetic parameters derived from the non-linear regression fit of experimental data, together with peptide sequences, are shown in (**b**). High micromolar *K*_*M*_ values indicate low affinity of HDAC6 for tested peptides, with limited contribution of residues that do not directly neighbor the central acetyllysine for the overall HDAC6 affinity/specificity. (**c**) Comparison of HDAC6 deacetylation rates using various substrates (natively-folded αβ-tubulin dimers, denatured tubulin dimers, peptides T3 through T19) at identical 3 μM concentrations. Deacetylation rates were determined using an HPLC assay for peptides and Western blotting quantification for tubulin dimers. Natively folded tubulin dimers are deacetylated 50- to 800-fold more efficiently than are either denatured tubulin dimers or “isolated” αK40 loop-derived peptides, suggesting that interactions/structural features outside the αK40 loop are required for efficient HDAC6/tubulin interactions. Data are presented as mean values ± s.d. (n = 3). (**d**) Steady-state kinetics of tubulin dimer deacetylation by HDAC6. Michaelis-Menten constants (K_M_ and *k*_*cat*_) were calculated from non-linear regression fit using the GraphPad program. Data represent mean values ± s.d. (n = 3).

To this end, we synthesized a series of peptides of different lengths derived from the human α-tubulin sequence that are centered around acetylated αK40 (T3–T19). Next, using a deacetylation assay followed by high-performance liquid chromatography (HPLC) analysis, we determined the kinetic parameters of HDAC6 against acetylated αK40-derived peptides (Fig. 3). The *K*_*M*_ values for the peptides were in the high micromolar range (88 µM to 328 µM for T9 and T15, respectively), revealing relatively low affinity of HDAC6 for the isolated αK40 sequences. At the same time, *K*_*M*_ values for all tubulin-derived peptides were very similar and there was no clear trend showing increasing affinity with the extension of the peptide sequence. Consequently, it is likely that within the isolated αK40 loop sequence, there is a limited contribution of residues beyond the P_1_ and P_−1_ positions to substrate binding/recognition. We also noticed a negative correlation between the peptide length and deacetylation rates, with the shortest T3 tripeptide being deacetylated 20-fold more efficiently when compared to the T19 sequence (Fig. 3a).

We then used the same experimental setup to directly compare deacetylation rates of the αK40 loop in its “free” peptidic form and in the context of αβ-tubulin dimers at a 3 µM concentration. Interestingly, free peptides were deacetylated approximately 12- to 200-fold less efficiently than were tubulin dimers (Fig. 3c). Additionally, we determined the kinetic parameters of HDAC6 for free tubulin to be *K*_*M*_ = 0.23 ± 0.05 µM and k_*cat*_ = 0.6 s^−1^ (Fig. 3d), revealing that the dramatically higher (>6,000-fold) HDAC6 catalytic efficacy (K_M_/k_cat_) for the natively-folded tubulin, as compared to free peptides, stems mostly from the increase in binding affinity, rather than from the increase in reaction speed. These results suggest that sequences and/or structural features outside the αK40 loop contribute to HDAC6/tubulin interactions. Such additional interaction sites might include lateral/longitudinal interfaces buried within tubulin polymeric lattices, as revealed by experiments where different MT polymers were used as substrates (see above).

### HDAC6 binds and deacetylates microtubules without a preference for microtubule tips

Given the relatively slow, but clearly distinguishable deacetylation of MTs by HDAC6, we asked how the enzyme accesses the αK40 loop in the MT lumen. Non-exclusive options would involve entering the MT lumen via open tips, through bends and breaks in the MT lattice, or by deacetylation of the αK40 loop “protruding” from the external microtubule face.

**Figure 4:**
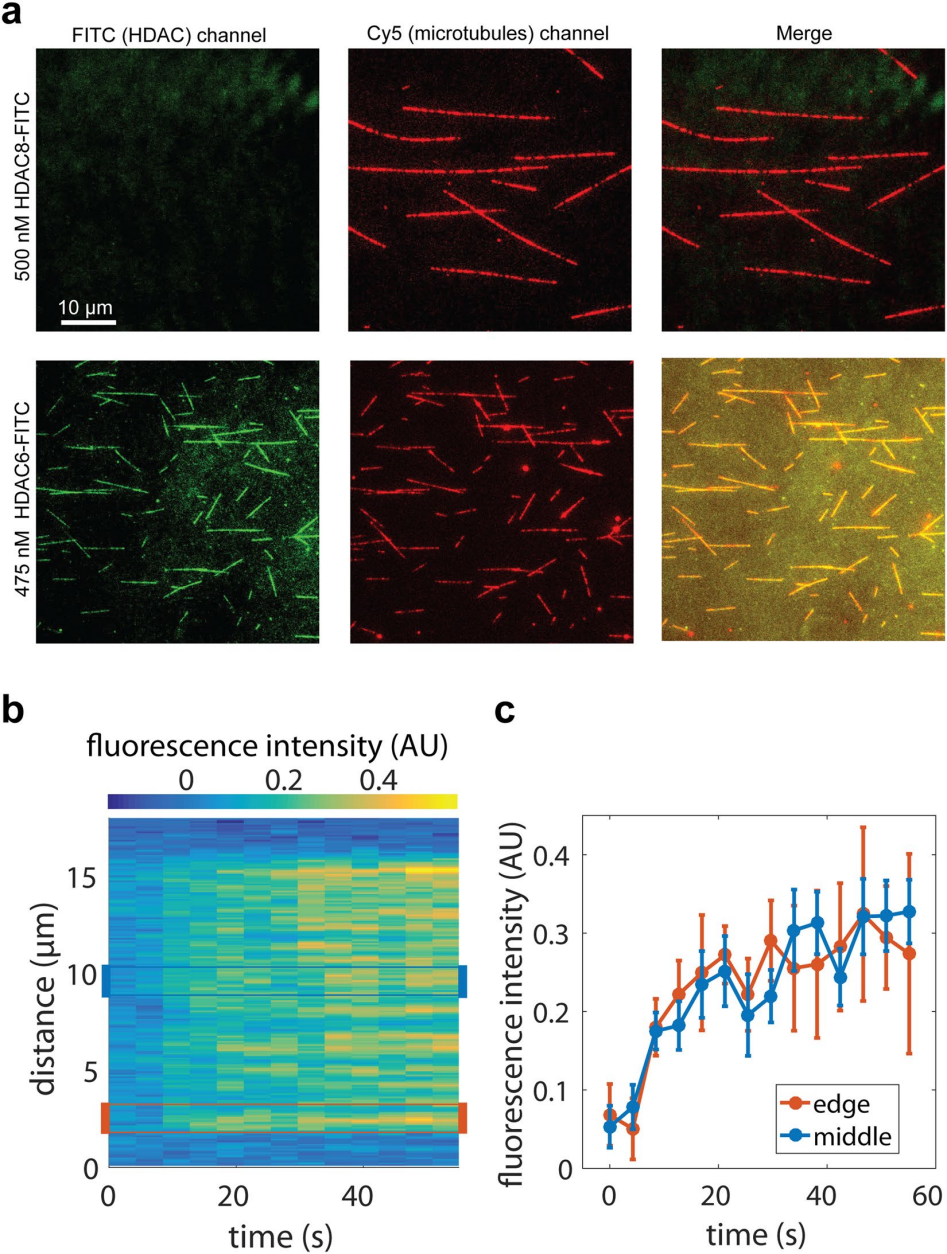
Interaction of HDAC6 with microtubules visualized by TIRF microscopy. (**a**) HDAC6 interacts directly with microtubules. Cy5-labeled microtubules were immobilized on a glass coverslip surface. Micrographs show microtubules (red) in the presence of 500 nM HDAC8-FITC (green; negative control, upper panel) or 475 nM FITC-labeled HDAC6 (green; lower panel). In the presence of FITC-labeled HDAC6, fluorescent signals of microtubules and HDAC6 co-localize along the whole length of microtubules (yellow). (**b**,**c)** HDAC6-GFP binds uniformly along the whole length of the microtubules. GFP-HDAC6 fusion was added to surface-immobilized microtubules and visualized in the 488 channel using TIRF microscopy. Uniform increase in GFP fluorescence intensity was observed along the whole length of microtubules. The kymograph in Panel B depicts averaged (background subtracted) GFP fluorescence intensity of 4 microtubules of the same length (approximately 12 µm) after the addition of HDAC6-GFP in the timespan from 0 to 60 seconds. The right panel shows the averaged GFP signal (±s.d.) over time at the edge of the microtubules (orange line) and in the middle (blue line). The regions (width of 1.5 µm) used for the averaging are indicated in the kymograph (**b**) by orange and blue rectangles.

To this end, we first investigated the localization and kinetics of HDAC6 interactions with assembled MTs using total internal reflection fluorescence (TIRF) microscopy. Rhodamine-labeled paclitaxel-stabilized MTs were immobilized on a cover slip, probed with either GFP-HDAC6, or FITC-HALO-HDAC6 fusions, and then directly visualized using a Nikon Ti-E microscope equipped with H-TIRF System (Fig. 4). The kinetics of HDAC6 binding to MTs were quite fast, with all MTs fully decorated in less than a minute. Based on signal distribution analysis during HDAC6 binding to the MTs, we did not detect any preferential binding to MT tips. Instead, we observed uniformly-distributed interactions along the MT length throughout the whole experiment, up until the time that the binding equilibrium was established. The rapid binding kinetics and even signal distribution suggest that HDAC6 does not enter the microtubule lumen from the open tips, but rather binds to and interacts with the external face of MTs.

**Figure 5:**
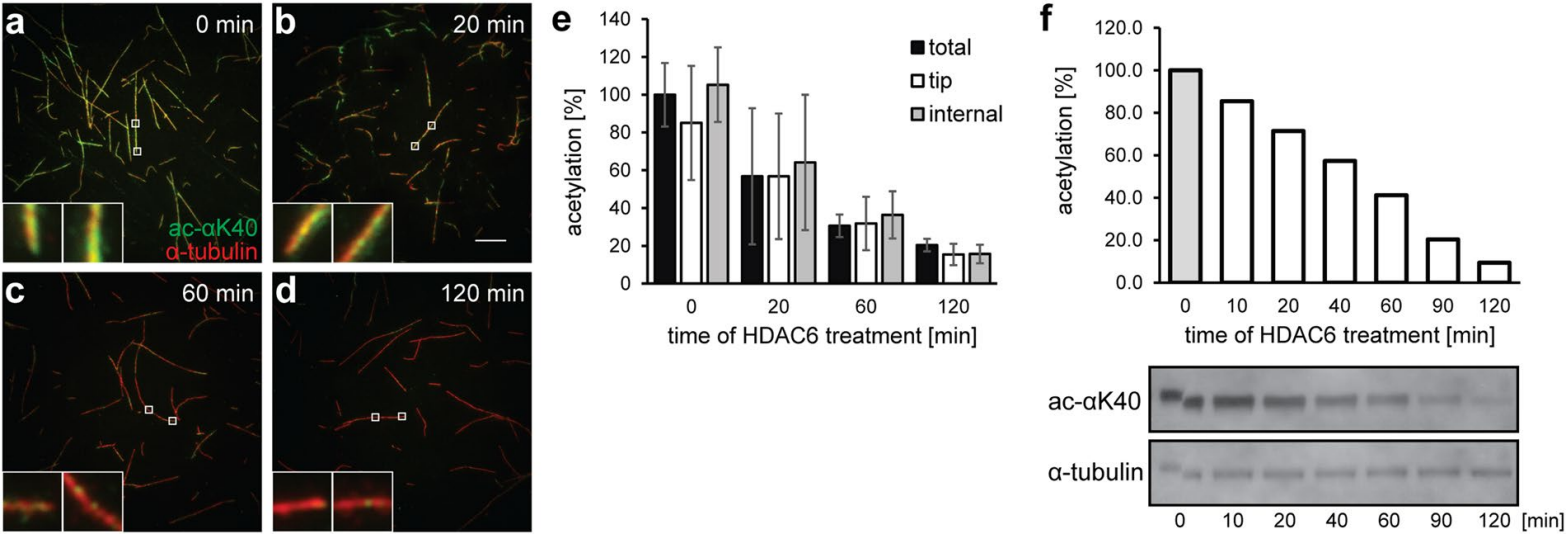
Stochastic, time-dependent deacetylation of assembled microtubules by HDAC6. *In vitro* assembled microtubules (1 µM) were treated with 2 µM human HDAC6 in BRB80 buffer. Deacetylation reactions were terminated at defined time points by the addition of 10 µM SAHA, and the acetylation of αK40 was quantified in parallel by indirect immunofluorescence microscopy (**a–e**) and Western blotting (**f**). (**a**–**e**) Indirect immunofluorescence microscopy was used to visualize the spatial distribution of αK40 acetylation signals along the length of microtubules at the given time points (upper right corner). Microtubules were immobilized on poly-L-lysine coverslips and probed with anti-αK(Ac)40 (green) and anti-α-tubulin (red) antibodies. The staining intensity of acetylated αK40 decreases over the time and the decrease parallels changes observed in the concomitant biochemical experiment. Additionally, the signal decrease is uniform over the whole length of the microtubules (see insets and panel e), suggesting that deacetylation of MTs by HDAC6 does not spread from the open tips, but is rather stochastic throughout the MT length. The left insets show microtubule tips and the right insets show the internal parts of microtubules positioned 10 µm from the tip. (**e**) Shows averaged intensity of the signal originating from a whole microtubule (total), the tip, and an area positioned 10 µm from the tip (internal). Bar = 10 µm. (**f**) Protein samples were electrotransferred to a PVDF membrane following SDS-PAGE separation, and then acetylation levels were quantified using an αK40-specific fluorescence signal normalized to the amount of total tubulin detected by rabbit polyclonal anti-α tubulin antibody. The decrease parallels changes observed in the concomitant immunofluorescence microscopy experiment.

Given the observed interactions of HDAC6 with the outer MT surface, we wondered whether HDAC6 binding to MTs is also translated into stochastic, rather than open-end favored, MT deacetylation. To answer this question, taxol-stabilized MTs (1 µM) were deacetylated using 2 µM HDAC6 in the BRB80 buffer, and the time course of deacetylation was then evaluated side-by-side both via fluorescence microscopy and biochemically (Fig. 5). The fluorescence microscopy and the parallel quantitative Western blotting revealed that the αK40 acetylation decreased over time, with only 10–15% of the original acetylation levels remaining after 120 minutes. More importantly, we observed that the extent of the deacetylation by HDAC6 was uniform along the whole MT length, as evidenced by the constant ratio of the immunofluorescence signal between the αK40 and total tubulin at microtubule tips, and the distance of 10 µm towards the MT center (Fig. 5a). Surprisingly, the spatio-temporal pattern of MT deacetylation appears to differ from the opposite reaction carried out by tubulin acetyltransferase, which favors open MT tips as the site of action^51–53^.

## Discussion

Although it has been known for more than a decade that HDAC6 is the primary deacetylase responsible for the removal of the acetylation mark at the αK40 position of tubulin, there are surprisingly few quantitative data characterizing human HDAC6 as tubulin deacetylase. Our report thus fills this gap by offering a detailed biochemical analysis of tubulin deacetylation by full-length human HDAC6, which reveals a strong preference for tubulin dimers over assembled microtubules. Additionally, we show that although isolated peptides derived from the αK40 loop are deacetylated by the enzyme, the structural context of native tubulin outside the loop is critical for highly efficient deacetylation. Finally, we directly visualized HDAC6/tubulin interactions for the first time, suggesting predominant HDAC6 binding to the external surface of assembled MTs.

The present study employed a bottom-up approach, where individual, isolated components were first purified and then combined and examined in a panel of biochemical and biophysical experiments. To this end, we cloned, expressed, and purified to homogeneity several human HDAC6 constructs, together with the corresponding control proteins in HEK293T cells. When we compared the activity of our HDAC6 preparations to the activities of recombinant proteins produced in *E. coli* or baculovirus systems (commercially available and/or reported in literature) our HEK293T-expressed enzymes were typically more active on equivalent substrates^42, 43, 48^. As the increased activity cannot be linked to post-translation modifications, which are absent from our samples, a more likely explanation would be a higher percentage of enzymatically competent molecules in our HDAC6 preparations, which are thus more suitable for the present biochemical experiments.

Next, we evaluated the *in vitro* HDAC6 activity using a panel of different substrates. At the peptide level, HDAC6 deacetylates αK40-derived peptides with relatively low affinity (high micromolar K_M_ values) and k_cat_ values in the range of 0.1–2 s^−1^. For comparison, SIRT2 deacetylates the T9 peptide somewhat less efficiently (k_cat_ = 0.144 ± 0.005 s^−1^ and k_cat_/K_M_ = 894 ± 100 M^−1^s^−1^)^17^, whereas αTAT1 has no activity on the T19 peptide^54^. The HDAC6 deacetylation of the T3–T19 peptide series clearly shows that amino acids beyond positions P_−1_ and P_+1_ around the central lysine are not critical for the recognition of the target peptidic substrates by HDAC6. These results are consistent with our unpublished data, as well as with reports mapping substrate specificity of HDAC6, which show that the enzyme is rather promiscuous at the peptide level, with few positional amino acid preferences^55^. A mechanistic explanation of these biochemical observations was recently provided by the crystal structure of HDAC6 complexes with tubulin/histone-derived peptides, which revealed that HDAC6/peptide contacts are limited to the recognition of the scissile acetyllysine, namely direct and water-mediated contacts with the acetyllysine amide and carbonyl groups, respectively^56^.

There is a clear difference between recognition and deacetylation of peptidic substrates and tubulin. Understandably, the recognition pattern of tubulin dimers by HDAC6 is considerably more complex than that of short peptides. This was supported in the current study by the substantially lower Michaelis constant (*K*_*M*_ = 0.23 µM) of the deacetylation reaction using tubulin dimers as a substrate (Fig. 3d). Additionally, overall catalytic efficacy (*k*_*cat*_ */K*_*M*_) was 2.6 µM^−1^s^−1^ and 0.00044 µM^−1^s^−1^ for tubulin dimers and the T19 peptide, respectively, revealing a striking 6,000-fold preference for native tubulin dimers as a substrate. Furthermore, in contrast to tubulin dimers isolated from porcine brains in a natively-folded state (confirmed by its ability to polymerize into MTs), when thermally-denatured tubulin was used as a substrate, its deacetylation rate by HDAC6 was much slower, more in the range of the deacetylation rates of free peptides (Fig. 3c), implying that the secondary/tertiary structural features, rather than simple linear amino acid motifs, outside the αK40 loop are critical for interactions with HDAC6. Our study points towards residues at the longitudinal/lateral interfaces of tubulin, which are buried in tubulin polymers, as being critical for free tubulin recognition/deacetylation. Although HDAC6/tubulin interactions are not fully understood, a study by Miyake *et al*., provided some mechanistic explanation by showing that in addition to residues located at the rim of the HDAC6 tunnel leading to the catalytic site, a uniquely-positioned H25 α-helix and Trp459 and Asn460 residues in a flexible loop joining helices H20 and H21 are critical for tubulin deacetylation. At the same time, substitutions at these positions have a limited effect on the deacetylation of peptidic substrates, indicating that the capacity to use/bind tubulin as a substrate, rather than the catalytic potential of HDAC6, is impaired^48^.

The quantitative data reported here demonstrate that free tubulin dimers are deacetylated by full-length human HDAC6 approximately 1,500-fold more efficiently than are assembled MTs. Similar preferences were also noticed by Miyake *et al*., who showed that the truncated *Danio rerio* enzyme prefers free tubulin over MTs, although in that study, the difference in the deacetylation rate was only approximately 2.5-fold. This paper, which is to the best of our knowledge the only other report providing quantitative data on HDAC6 preferences for individual tubulin forms, suggests that there are substantial differences in tubulin deacetylation among individual HDAC6 orthologs and likely also between full-length proteins and constructs truncated beyond the tandem catalytic domains. Detailed studies concerning the impact of these and other variables (presence/absence of accessory proteins, post-translational modifications on HDAC6/tubulin) will be required to understand and reconcile intriguing discrepancies in HDAC6 substrate preferences (from an absolute preference for MTs to favoring free heterodimers) that have been reported in prior *in vivo/in vitro* studies^15–17, 38, 42, 48, 57^.

Although HDAC6 is the major tubulin deacetylase, interactions between MTs and HDAC6 have not yet been visualized. Our study thus offers the first direct visualization of HDAC6 binding to the MT surface. The reported flush-in experiment, where the chamber with surface-immobilized MTs was injected with the GFP-HDAC6 fusion and binding kinetics quantified by TIRF microscopy, reveals that kinetics of HDAC6 binding to MTs are relatively fast. The initial increase in the fluorescence signal was observed within 10 seconds following the flush-in and the signal plateau was reached approximately 30 seconds later (Fig. 4). Such kinetics, together with the even distribution of the signal along the whole MT length within the monitored time period, strongly suggest that HDAC6 binds to the outer MT surface rather than diffusing into the MT lumen via open tips. This observation does not formally exclude the possibility of HDAC6 entering and binding/functioning within the lumenal cavity of MTs, but this would likely happen with considerably slower kinetics. It should also be noted that HDAC6 deacetylase activity is not required for HDAC6 binding to MTs, as the TIRF assay in the presence of the HDAC6 inhibitor Nexturastat A gave virtually indistinguishable binding curves (data not shown).

Biochemical or physiological implications of HDAC6 binding to the external surface of MTs are unclear at present. For example, such interactions can help with targeting and concentrating the enzyme at the site of its action, and binding can help HDAC6 ﻿to access the αK40 either via the open MT ends and/or through fluctuations/defects in the MT lattice observed in previous studies^58–60^. Alternatively, the binding of HDAC6 can influence the stability of MTs^61, 62^ and/or the dynamic properties of MTs^16^. Further structural and biological studies are required to provide more insight into these outstanding issues.

It has been shown that αTAT1 enters MT lumen in order to acetylate αK40^2, 51, 63^, and that acetylation starts at the open tips of MTs, followed by the acetylation mark progressively spreading by longitudinal diffusion of αTAT1 in the MT lumen. Alternatively, αTAT1 can enter MT lumen via structural defects in the MT lattice. Irrespective of the mode of αTAT1 entry into the MT lumen, assembled MTs are a preferred substrate for αTAT1^51–53^. In contrast, our study reveals stochastic deacetylation of MTs by HDAC6 without any tip preferences (Fig. 5), and a similar deacetylation pattern was reported for the tandem catalytic domain construct of *D. rerio* HDAC6^48^. The underlying cause for spatial differences in αK40 acetylation and deacetylation reactions by αTAT1 and HDAC6, respectively, remains unknown at present.

**Figure 6:**
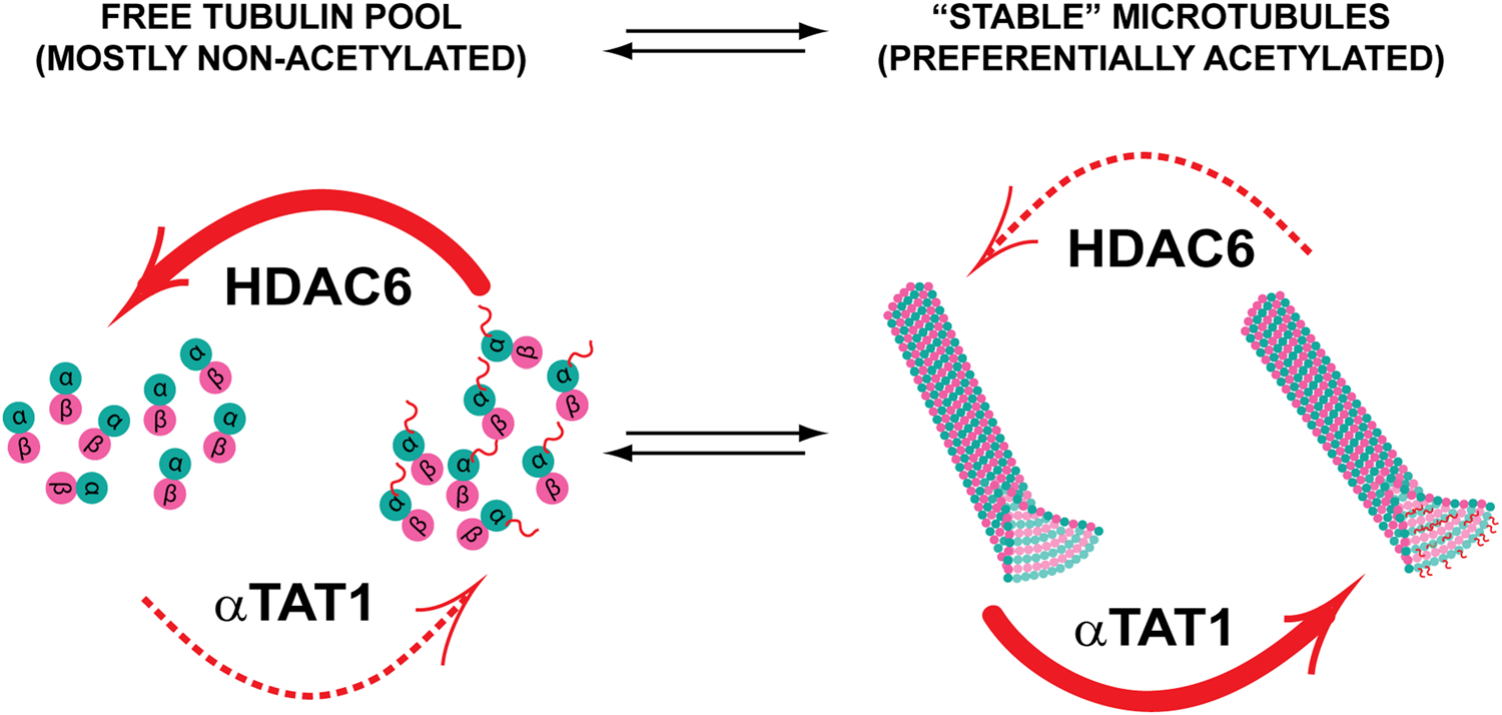
High acetylation levels of stable microtubules are linked to opposing substrate preferences of HDAC6 and αTAT1. HDAC6 prefers free tubulin as a substrate, whereas αTAT1 is 5-fold more active on assembled microtubules (preferences shown as thick red arrows). Consequently, it is likely that within the cell, the pool of free tubulin is mostly in the non-acetylated state. Once microtubules are assembled, however, they become preferentially acetylated by αTAT1. For dynamic microtubules, their lifetime may be too short to become extensively acetylated, whereas stable microtubules with long lifespans have a much higher probability of being fully acetylated by αTAT1. Both HDAC6 and αTAT1 thus serve as “timers” to set the clocks gauging microtubule age based on their acetylation status.

Overall, our study adds another piece of information to the complex puzzle of how tubulin acetylation status is controlled by the opposing activities of αTAT1 and HDAC6, as previously suggested by others^48, 64^. To this end, our data complement these findings concerning the αTAT1/tubulin interplay, and provide further rationale for the correlation between tubulin acetylation and microtubule age. As HDAC6 prefers free tubulin dimers with a velocity of >0.6 mol/mol*s, it is tempting to speculate that the pool of free tubulin dimers within cells should be mostly non-acetylated. Once MTs are formed, however, these become preferred substrates for αTAT1 and are therefore preferentially acetylated. At the same time, MTs are only inefficiently deacetylated by HDAC6. Consequently, given the inverse preferences of HDAC6 and αTAT1 for free tubulin and MTs, respectively, in the context of long-lived stable MTs, the equilibrium is clearly shifted towards acetylated αK40 (Fig. 6). Finally, ongoing investigations in our laboratory are focused on building up the complexity of our experimental system by evaluating certain variables encountered *in vivo*, such as post-translational modifications, HDAC6/tubulin interaction partners, and MT dynamics, with the aim of describing and dissecting their individual contributions and mutual interplay in relation to tubulin deacetylation by HDAC6.

## Methods

### Chemicals and peptides

If not stated otherwise, all chemicals were purchased from Sigma-Aldrich (St. Louis, MO, USA). Peptides were synthesized commercially by KareBay (Monmouth Junction, NJ, USA) at >90% purity and their identities were confirmed by analytical LC-MS (data not shown).

### Expression plasmids

The sequence encoding human HDAC6 (NP_006035.2, UniProtKB - Q9UBN7) was used as a template for cloning all HDAC6 variants used in this report. In general, a nucleotide sequence encoding a given variant was PCR amplified with a set of desired primer pairs and inserted into the pDONR221 plasmid using the BP recombination reaction (Invitrogen, Carlsbad, CA, USA), according to the manufacturer’s protocol. The identity of the resulting entry clone was confirmed by Sanger sequencing. Expression plasmids were generated via LR recombination reaction between the entry clone and a required destination vector, which typically introduced a TEV-cleavable tag at the N-terminus of the HDAC6 variant to simplify purification and/or visualization of the resulting fusion. Schematic representations of constructs used in this study are shown in Supplementary Fig. S1.

### Large-scale expression of HDAC6 variants

All HDAC6 variants were expressed using HEK-293/T17 cells following transient transfection mediated by linear polyethylene imine (PEI) (Polysciences Inc., Warrington, PA, USA). To this end, the suspension culture of HEK-293/T17 cells was grown in 2 L Erlenmeyer flasks in Free Style F17 medium (Thermo Fisher Scientific) supplemented by 0.1% Pluronic F-68 (Invitrogen) and 2 mM L-glutamine at 110 rpm under a humidified 5% CO_2_ atmosphere at 37 °C. For large-scale expression, 0.7 mg of an expression plasmid was diluted in 17.5 ml of PBS, to which 2.1 ml of 1 mg/ml PEI was added. The mixture was vortexed briefly, incubated for 10 min at room temperature, and then added to 350 ml of cells at a concentration of 4 × 10^6^ cell/ml. Four hours post-transfection, the cell suspension was diluted by an equal volume of ExCell serum-free medium. Cells were harvested 72 h post transfection by centrifugation at 500 × g for 5 min, then the cell pellet was frozen in liquid nitrogen and stored at −80 °C until further use.

### Purification of HDAC6 variants

The cell pellet was lysed by sonication (24 W/1 min) in ice-cold lysis buffer (100 mM Tris-HCl, 10 mM NaCl, 5 mM KCl, 2 mM MgCl_2_, 10% glycerol; pH 8.0) supplemented with benzonase (1 U/ml; Merck, Darmstadt, Germany) and protease inhibitor coctail (Roche, Basel, Switzerland). To further assist cell lysis, Igepal-630 was added to the cell lysate to a final concentration of 0.2% (v/v) and the mixture was incubated for 20 min on ice. NaCl was added to a final concentration of 150 mM. The lysate was then cleared by centrifugation at 40,000 × g for 30 min at 4 °C and the supernatant was loaded onto a Strep-Tactin column (IBA, Gottingen, Germany) equilibrated in the lysis buffer. Following the washing step with the lysis buffer, the fusion protein was eluted using 50 mM Tris-HCl, 150 mM NaCl, 10 mM KCl, 10% glycerol and 3 mM desthiobiotin (pH 8.0). The eluted fusion protein was concentrated to approximately 1 mg/ml and (if desired) the N-terminal tag was cleaved by the addition of the 1:20 (w/w) TEV protease overnight at 4 °C. The HDAC6 protein was then separated from tags and the TEV protease by size exclusion chromatography using the Superdex 16/600 HR200 column (GE Healthcare Bio-Sciences, Little Chalfont, UK) with 30 mM HEPES, 140 mM NaCl, 10 mM KCl, 3% glycerol, and 0.25 mM TCEP (AMRESCO, Solon, OH, USA), with a pH 7.4 adjusted with NaOH, as a mobile phase. The purified protein was concentrated to approximately 1 mg/ml and aliquots were flash frozen in liquid nitrogen and stored at −80 °C until further use.

### Fluorometric assays

Deacetylation activity of HDAC6 variants was determined using acetyl-Gly-Ala-(acetyl-Lys)-AMC (GAK(Ac) -AMC; Bachem, Bubendorf, Switzerland), Boc-(acetyl-Lys)-AMC (Boc-K(Ac)-AMC; Bachem) and Fluor-de-Lys (Enzo Life Sciences, Plymouth Meeting, PA, USA) substrates. In general, a given HDAC6 variant was incubated with a substrate in a reaction buffer comprised of 50 mM HEPES, 140 mM NaCl, 10 mM KCl, 1 mg/ml bovine serum albumin (BSA), and 1 mM TCEP, at a pH 7.4 adjusted with NaOH (total volume 20 µl). This incubation occurred in 384-well plates for 30 min with vigorous shaking at 37 °C. The reaction was stopped by addition of 20 µl of trypsin solution (2 mg/ml trypsin, 20 mM Tris-HCl, 150 mM NaCl, 1 mM EDTA; pH 7.4 adjusted with NaOH). Following the 15-min incubation at 37 °C, a fluorescence signal of released aminomethylcoumarin was quantified using a CLARIOstar fluorimeter (BMG Labtech GmbH, Ortenberg, Germany) with excitation/emission wavelengths set at 365/440 nm, respectively. The data were fitted using the GraphPad Prism software (GraphPad Software, San Diego, CA, USA) and kinetic values were calculated by non-linear regression analysis.

### HPLC-based assays

Individual fluorophore-labeled peptides were incubated with HDAC6 at 37 °C for 30 min in an assay buffer comprised of 50 mM HEPES, 140 mM NaCl, 10 mM KCl, 2 mg/ml BSA, and 1 mM TCEP, at a pH 7.4 adjusted with NaOH. The reaction was quenched by the addition of acetic acid to a final concentration of 0.5%, and reaction products were quantified by means of RP-HPLC (Shimadzu, HPLC Prominence system) on the column Kinetex® 2.6 µm XB-C18 100 Å, 100 × 3 mm (Phenomenex, Torrance, CA, USA).

### Determination of inhibition constant

The inhibition constants for SAHA (Selleckchem, Houston, TX, USA), Trichostatin A, and Nexturastat A (gift from A. Villagra and A. Kozikowski from George Washington University, USA, and University of Illinois at Chicago, USA, respectively) were determined using the slightly modified fluorogenic assay described above. Briefly, the tested compounds were preincubated with 0.3 nM human recombinant HDAC6 at 37 °C for 15 min, and then the deacetylation reaction was started by addition of 10 µM GAK(Ac)-AMC. After a 120-min incubation at 37 °C, the reaction was stopped by the addition of trypsin solution. Following the 15-min incubation at 37 °C, a fluorescence signal of released aminomethylcoumarin was quantified using a CLARIOstar fluorimeter with excitation/emission wavelengths set at 365/440 nm, respectively. The data were fitted using the GraphPad Prism software and IC_50_ values were calculated by non-linear regression analysis. The inhibitor and enzyme-free controls were defined as 100% and 0% HDAC6 activity, respectively.

### Isolation of tubulin from porcine brains

Tubulin isolation from pig brain tissue was carried out according to an established protocol (details in the supplementary material)^65^. The concentration of the final tubulin preparation was determined based on the absorbance at 280 nm. Supernatant containing purified tubulin was flash frozen in aliquots using liquid nitrogen and stored at −80 °C.

### Determination of αK40 acetylation levels

Five µg of porcine tubulin were diluted in 100 µl of 50 mM ammonium bicarbonate (pH 8.5) and then digested with GluC (V8) protease (Roche) for 10 hours at a ratio of 1:100. After complete evaporation on speedvac, the sample was dissolved in 15 µl of 0.1% formic acid, 10% dimethylsulfoxide in water. Acetylated and non-acetylated versions of the peptide HGIQPDGQMPSDKTIGGGDDSFNTFFSE with isotopically-labeled isoleucine (heavy peptides) was added into the mixture of the same concentration. Three µl of the sample were analyzed on the UltiMate 3000 RSLCnano system (Dionex, Sunnyvale, CA, USA) coupled to a TripleTOF 5600 mass spectrometer with a NanoSpray III source (AB Sciex, Framingham, MA, USA). The peptides were trapped and desalted with 2% acetonitrile in 0.1% formic acid at a flow rate of 5 μl/min on the Acclaim PepMap100 column (5 μm, 2 cm × 100 μm ID, Thermo Scientific). Eluted peptides were separated by the Acclaim PepMap100 analytical column (3 μm, 25 cm × 75 μm ID, Thermo Scientific) using a 70-min elution gradient at a constant flow of 300 nl/min, with mobile phase A being 0.1% formic acid and mobile phase B being 0.1% formic acid in acetonitrile. Extracted ion chromatogram of the αK40 containing identified peptides, either acetylated or nonacetylated, in light and heavy forms, were generated and the area of relevant peaks of charge state 3 was recorded. The area of light peptides was normalized to the heavy peptide area, and the percentage of acetylated versus non-acetylated peptides was calculated.

### Labeling of HALO-HDAC6 fusions

The HALO-HDAC6 variants were labeled via covalent modification of the HALO fusion partner using the HaloTag-FITC ligand. The HALO-HDAC6 fusion (1 mg/ml) was incubated with a 5 molar excess of the ligand overnight at 4 °C. The unbound labels were removed by size exclusion chromatography using the Superdex 10/300 HR200 column with 30 mM HEPES, 140 mM NaCl, 10 mM KCl, 3% glycerol, and 0.25 mM TCEP, at pH 7.4 adjusted with NaOH, as a mobile phase, and labeled fusions were concentrated to approximately 1 mg/ml, aliquoted, and then snap-frozen in liquid nitrogen.

### Preparation of tubulin polymers

To obtain taxol-stabilized microtubules, tubulin at a concentration of 4.4 mg/ml was polymerized for 30 min at 37 °C in BRB80 buffer supplemented with 4.8% DMSO, 4 mM MgCl_2_, and 1 mM GTP, after which BRB80 with 20 µM paclitaxel was added. For GMPCPP-stabilized microtubules, tubulin at a concentration of 0.25 mg/ml was incubated for 2 h at 37 °C in BRB80 buffer supplemented with 1 mM MgCl_2_ and 1 mM GMPCPP (Jena Bioscience, Jena, Germany). Dolastatin-10 rings resulted from the polymerization of 2.2 mg/ml tubulin for 40 min at room temperature with 40 µM dolastatin-10 in 80 mM PIPES (pH 6.9), 50 mM KCl, 1 mM EGTA, 1 mM MgCl_2_, and 1 mM DTT. After the polymerization, all abovementioned polymers were pelleted at 30,000 × g for 40 min at 37 °C and the pellet was suspended in warm BRB80 with 20 µM taxol to the required concentration. Zn-sheets were prepared via 2 h incubation of 3 mg/ml tubulin at 37 °C in 80 mM MES, 200 mM NaCl, 3 mM GTP, 1.25 mM MgSO_4_, 1.25 mM ZnSO_4_, and 0.025 mg/ml pepstatin, adjusted to a pH of 5.5 with NaOH. Zn-sheets were then stabilized by the addition of paclitaxel to a final concentration of 31.5 µM. Zn-sheets were pelleted at 30,000 × g for 40 min at 37 °C and the pellet was suspended in a warm solution of 80 mM MES, 200 mM NaCl, 1.25 mM MgSO_4_, and 20 µM taxol, adjusted to a of pH 5.5 with NaOH.

### Microtubule binding assay

Microtubules and flow cells were prepared as described previously^66^. Briefly, taxol-stabilized microtubules were polymerized from tubulin purified from pig brains using a 1:30 ratio of Alexa-647-labeled tubulin to unlabeled tubulin, at a concentration of 2 µM and 1 mM GMPCPP in BRB80. Microscope chambers were constructed of silanized coverslips, with parafilm used to space them to form channels of 0.1-mm thickness, 3-mm width, and 18-mm length. Silanization was performed as described previously^67^. Microtubules were immobilized to the glass surface of a flow cell covered with anti-β-tubulin antibodies (Sigma-Aldrich, St. Louis, MO, USA, #T7816, 20 µg/ml in PBS), and then GFP-labeled HDAC6 was flushed into the flow cell at a final concentration of 300 nM. All experiments were carried out in a buffer consisting of BRB50, 1 mM TCEP, 0.5 mg/ml casein, 10 µM paclitaxel, 20 mM D-glucose, 110 µg/ml glucose oxidase, and 20 µg/ml catalase.

### TIRF microscopy

Binding experiments were visualized using TIRF microscopy. Experiments were carried out using a Nikon Ti-E microscope equipped with H-TIRF System, 60x oil immersion 1.49 NA TIRF objective and Andor Ixon Ultra EMCCD camera (Andor Technology, Belfast, UK) controlled by NIS Elements software (Nikon). Alexa-647-labeled microtubules and GFP-labeled HDAC6 in microtubule binding assays were visualized sequentially by switching between 640 nm and 488 nm excitation lasers. The image acquisition rate was 0.5 frames per second for sequential dual-color imaging, and image analysis was performed using Fiji^68^ and MatLab (MathWorks).

### Deacetylation of tubulin dimers and polymers

Using a fluorescence peptide-based assay, we first verified that small molecule components (e.g., taxol, GMPCPP, Dolastatin-10, Zn^2+^ ions) do not interfere with HDAC6 deacetylase activity at levels above the highest concentrations used in our assays (data not shown). Deacetylation reactions in a total volume of 20 µl were performed in BRB80 buffer (experiments with Zn sheets were performed in 80 mM MES, 200 mM NaCl, and 1.25 mM MgSO_4_, adjusted to pH 5.5 with NaOH) supplemented with 1 mM TCEP and 0.5 mg/ml BSA, at 37 °C for 30 min in the case of dimers; for microtubules, the reaction was continuous and aliquots were usually harvested at 0.5, 1, 2, and 3 h. Prior to the reaction, HDAC6 was diluted to the required concentrations in the BRB80 buffer supplemented with TCEP and BSA, and the reaction was started by the addition of tubulin dimers/microtubules. Colchicine (40 µM) and paclitaxel (10 µM) were added to reaction mixtures of free tubulin dimers and microtubules, respectively. For assays with denatured tubulin dimers, the mixture of free tubulin dimers was incubated for 20 min at 95 °C, then cooled and deacetylated as native substrates. The deacetylation reaction was stopped by the addition of 20 µl of 2x Laemmli buffer and boiling samples at 95 °C for 5 min.

### SDS-PAGE, Western blotting, and data analysis

Samples were loaded onto a 4–20% gradient PAGE gel (GenScript, Piscataway, NJ, USA) at 150 ng of tubulin per lane, and then run in MOPS-SDS running buffer at 160 V for 45 mins. Gels were electrotransferred onto a PVDF membrane that was subsequently blocked with 5% (w/v) BSA in TBS. The level of tubulin acetylation at αK40 was determined using a monoclonal anti-acetylated tubulin antibody^69^ (Sigma, T7451, 0.3 µg/ml); a secondary goat anti-mouse antibody conjugated to Alexa Fluor 488 (Life Technologies, A11029, dilution 0.4 µg/ml) and normalized to the amount of total tubulin detected by rabbit polyclonal anti-α tubulin antibody (Abcam, Cambridge UK, ab18251, 1 µg/ml); and a secondary donkey anti-rabbit antibody conjugated to Alexa Fluor 568 (Life Technologies, USA, A10042, 0.4 µg/ml). HDAC6 was visualized using a custom-made anti-HDAC6 polyclonal rabbit sera at 1:5,000 dilution. Fluorescence intensity was measured by Typhoon FLA9500 imager (GE Healthcare Bio-Sciences) and quantified using Quantity One 1-D Analysis Software (Bio-Rad, Hercules, CA, USA). Data analysis of enzyme kinetics and statistical analysis using one-way ANOVA with Tukey’s post hoc tests were performed using Graph Pad Prism software.

### Indirect immunofluorescence microscopy

Deacetylation experiments were also visualized using indirect immunofluorescence microscopy. Deacetylation reactions were terminated by the addition of 10 µM SAHA, and the mixture of microtubules and HDAC6 was applied to a glass slide pretreated by poly-L-lysine. The following steps were carried out in PBS supplemented with 10 µM taxol at room temperature. Coverslips were washed with PBS and blocked with 0.5% BSA for 20 min. Coverslips were then incubated with anti-acetylated tubulin monoclonal antibody (10 µg/ml) and rabbit polyclonal anti-α tubulin antibody (2.5 µg/ml) for 20 min. Following the washing step, goat anti-mouse antibody conjugated to Alexa Fluor 488 and goat anti-rabbit antibody conjugated to Alexa Fluor 594 were applied for 20 min. After the final wash, coverslips were mounted in VectaShield medium (Vector Laboratories), and images were obtained using the Nikon Eclipse Ti fluorescence microscope equipped with a 100x immersion oil objective and additive 2.5x lens magnification (Nikon), as well as with the ORCA-flash 4.0 digital CMOS camera (Hamamatsu Photonics, Japan). Images were processed using Adobe Photoshop software and the signal intensity was quantified using Fiji.

### Data availability

All data generated or analysed during this study are included in this published article (and its Supplementary Information files).

## Electronic supplementary material

Supplementary Material
