# Supplementary material for: Human histone deacetylase 6 shows strong preference for tubulin dimers over assembled microtubules
Source: Sci Rep. 2017 Sep 14;7:11547. doi: 10.1038/s41598-017-11739-3 (PMC5599508; doi:10.1038/s41598-017-11739-3)
Supplement: Supplementary file 1 — Supplementary Material [file 41598_2017_11739_MOESM1_ESM.pdf]

Supplementary Material

Human histone deacetylase 6 shows strong preference for tubulin dimers over assembled microtubules

Lubica Skultetyova[1,2], Kseniya Ustinova[1], Zsofia Kutil[1], Zora Novakova[1], Jiri Pavlicek[1], Jana Mikesova[1], Dalibor Trapl[1], Petra Baranova[1], Barbora Havlinova[1], Martin Hubalek,[3] Zdenek Lansky[1] and Cyril Barinka[1]*

[1]Institute of Biotechnology CAS, BIOCEV, Prumyslova 595, 252 50 Vestec, Czech Republic
[2]Department of Biochemistry, Faculty of Natural Science, Charles University, Albertov 6, Prague 2, Czech Republic
[3]Gilead Sciences and IOCB Research Center, Institute of Organic Chemistry and Biochemistry of the Academy of Sciences of the Czech Republic, Flemingovo n. 2, 166 10 Prague 6, Czech Republic.

Supplementary M&M:

Isolation of tubulin from porcine brains
Ten fresh pig brains were homogenized in DB buffer (50 mM MES, 1 mM $CaCl_2$, pH 6.6 adjusted with NaOH) at 4°C using a blender (Waring Commercial, Stamford, CT, USA). Homogenate was centrifuged at 29,000xg for 1 hour at 4°C and supernatant mixed with HMP buffer (1 M PIPES, 10 mM $MgCl_2$, 20 mM EGTA, pH 6.9 adjusted with KOH) and glycerol in volume ratio 1:1:1 and supplemented with ATP (Jena Bioscience, Jena, Germany), and GTP (Jena Bioscience), to a final concentration 1.5 mM and 0.5 mM, respectively. The mixture was incubated for 1 hour at 37°C and then centrifuged at 151,000xg for 30 minutes at 37°C. The pellet was resuspended in 100 ml ice-cold DB buffer and homogenized by a teflon-glass manual homogenizer for 10 minutes on ice, incubated for 30 minutes on ice and then centrifuged at 70,000xg for 30 minutes at 4°C. Supernatant was mixed with HMP buffer and glycerol in volume ratio 1:1:1 and supplemented with ATP and GTP to a final concentration 1.5 mM and 0.5 mM, respectively. Mixture was incubated for 30 minutes at 37°C and centrifuged at 151,000xg for 30 minutes at 37°C. Pellet was suspended in 10 ml BRB80 buffer (80 mM PIPES, 1 mM $MgCl_2$, 1 mM EGTA, pH 6.8 adjusted with KOH), homogenized by teflon-glass manual homogenizer for 10 minutes on ice, incubated 10 minutes on ice, and centrifuged at 100,000xg for 30 minutes at 4°C. Supernatant containing purified tubulin was flash frozen in aliquots by liquid nitrogen and stored at -80°C.

Supplementary Figures

Fig. S1: Protein constructs used in this study. A; Schematic representation of full-length human HDAC6, its N-terminal fusions and the control GFP-HALO fusion. HDAC6 constructs and the GFP-HALO fusion were produced in suspension HEK293T cells, and purified to homogeneity *via* optimized protocols using the combination Streptactin affinity and size-exclusion chromatography. TEV – TEV-protease recognition site; Strep – Strep-tag; FLAG – FLAG tag. B; Coomassie-stained SDS-PAGE of purified HDAC6 variants. Purity of all protein versions was >98%. C; Steady-state kinetics of HDAC6, GFP-HDAC6 and HALO-HDAC6 on commercial fluorogenic peptide (Ac)GAK(Ac)-AMC. Michaelis-Menten constants (K_M and k_{cat}) for individual construct, calculated from non-linear regression fit using the GraphPad program, are shown in the embedded table.

Fig. S2: Determination of αK40 acetylation ratio

A; The extracted ion chromatogram of $[M+3H]^{3+}$ ions of light (m/z = 995.44) and heavy (m/z = 997.78) non-acetylated version of peptide HGIQPDGQMPSDKTIGGGDDSFNTFFSE. **B**; The extracted ion chromatogram of $[M+3H]^{3+}$ ions of light (m/z = 1009.44) and heavy (m/z = 1011.78) acetylated version of peptide HGIQPDGQMPSDK(Ac)TIGGGDDSFNTFFSE. **C**; Area of corresponding peaks with calculation of acetylation ratio.

Fig. S3: HDAC6 reveals significantly higher deacetylase activity on tubulin dimers than on polymeric tubulin forms.

Uncropped images of representative Western blots shown in Figure 2 of the main manuscript body.

Figure S1

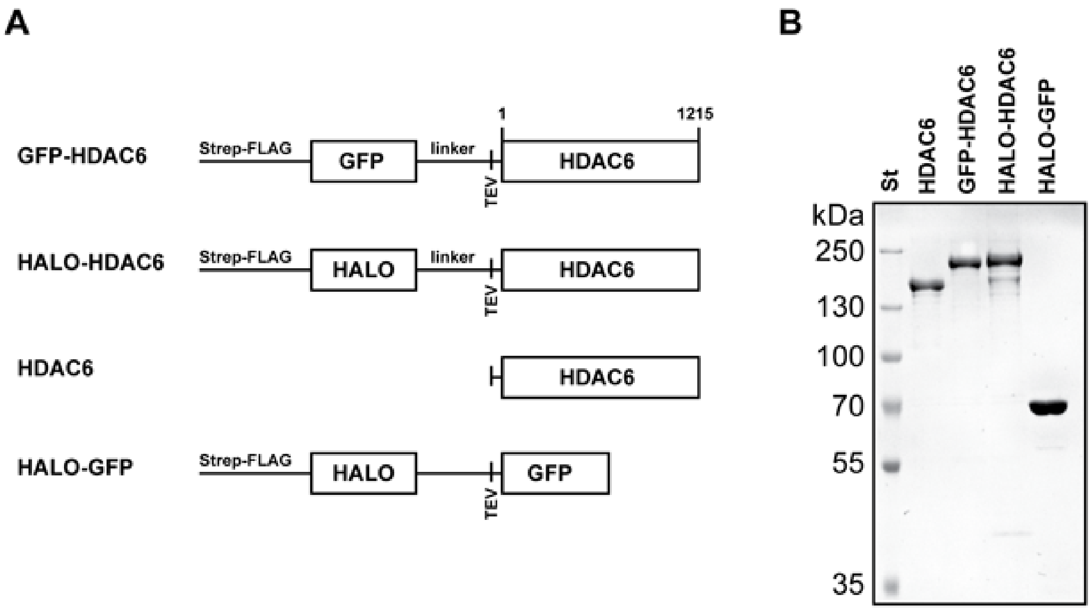

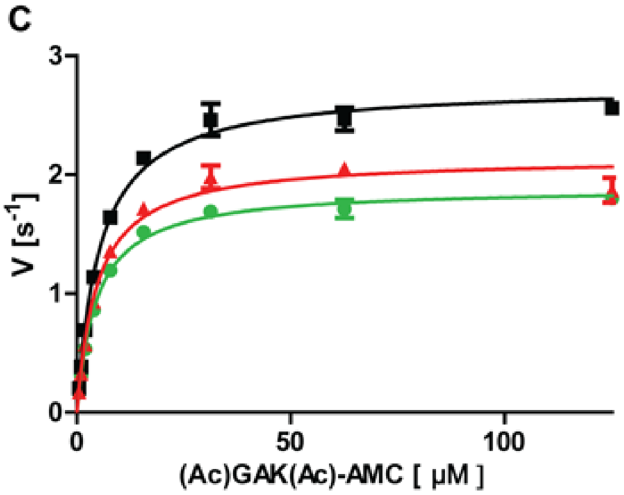

	Construct	K_M [μM]	k_{cat} [s^{-1}]
▲	HALO-HDAC6	5.3 ± 0.4	2.75 ± 0.05
▼	GFP-HDAC6	4.8 ± 0.5	2.15 ± 0.06
■	HDAC6	4.7 ± 0.3	1.89 ± 0.03

Figure S2

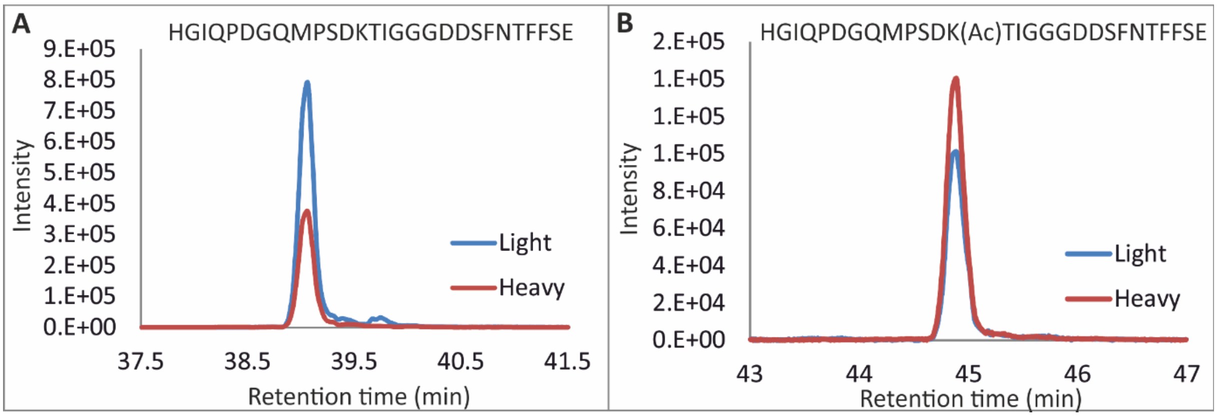

C

		Area	L/H ratio
αK40 NonAc	light	8.47E+06	2.13
	heavy	3.97E+06	
αK40 Ac	light	1.26E+06	0.76
	heavy	1.66E+06	

Ac/NonAc ratio = 0,36

Figure S3

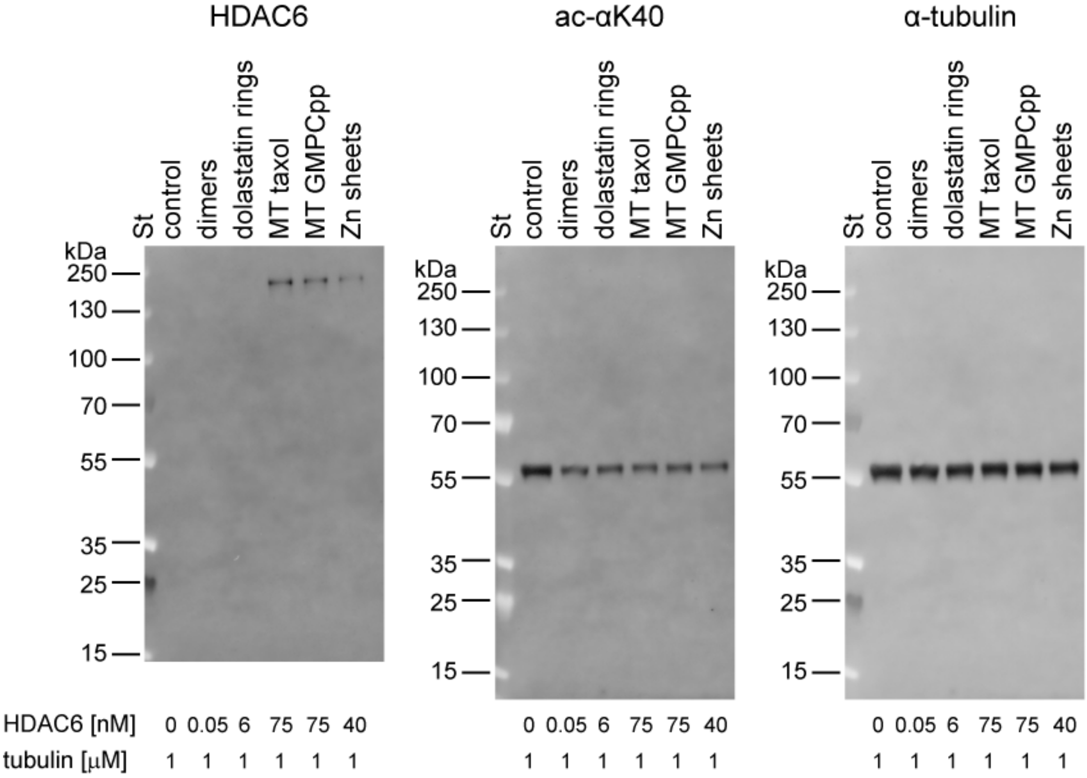
HDAC6
ac-αK40
α-tubulin
St
control
dimers
dolastatin rings
MT taxol
MT GMPCpp
Zn sheets
kDa
250
130
100
70
55
35
25
15
HDAC6 [nM] 0 0.05 6 75 75 40
tubulin [μM] 1 1 1 1 1 1
